# Image Matching: Foundations, State of the Art, and Future Directions

**DOI:** 10.3390/jimaging11100329

**Published:** 2025-09-24

**Authors:** Ming Yang, Rui Wu, Yunxuan Yang, Liang Tao, Yifan Zhang, Yixin Xie, Gnana Prakash Reddy Donthi Reddy

**Affiliations:** 1 Department of Information Technology, Kennesaw State University, Kennesaw, GA 30060, USA; rwu3@kennesaw.edu (R.W.); ltao1@students.kennesaw.edu (L.T.); yxie11@kennesaw.edu (Y.X.); gdonthi1@students.kennesaw.edu (G.P.R.D.R.); 2 Department of Electrical Engineering, Columbia University, New York, NY 10027, USA; yy3526@columbia.edu; 3 Department of Computer Science, Missouri State University, Springfield, MO 65897, USA; yifanzhang@missouristate.edu

**Keywords:** image matching, feature points, SIFT, SURF, SuperPoint, SuperGlue, LoFTR

## Abstract

Image matching plays a critical role in a wide range of computer vision applications, including object recognition, 3D reconstruction, aiming-point and six-degree-of-freedom detection for aiming devices, and video surveillance. Over the past three decades, image-matching algorithms and techniques have evolved significantly, from handcrafted feature extraction algorithms to modern approaches powered by deep learning neural networks and attention mechanisms. This paper provides a comprehensive review of image-matching techniques, aiming to offer researchers valuable insights into the evolving landscape of this field. It traces the historical development of feature-based methods and examines the transition to neural network-based approaches that leverage large-scale data and learned representations. Additionally, this paper discusses the current state of the field, highlighting key algorithms, benchmarks, and real-world applications. Furthermore, this study introduces some recent contributions to this area and outlines promising directions for future research, including H-matrix optimization, LoFTR model speedup, and performance improvements. It also identifies persistent challenges such as robustness to viewpoint and illumination changes, scalability, and matching under extreme conditions. Finally, this paper summarizes future trends for research and development in this field.

## 1. Introduction

### 1.1. What Is Image Matching?

Image matching is a fundamental problem in computer vision that involves finding correspondences between two or more images of the same scene, object, or structure. It serves as a critical building block for numerous high-level tasks, such as image stitching, 3D reconstruction, simultaneous localization and mapping (SLAM) [1], object recognition, aiming-point and six-degree-of-freedom (6DOF) detection [2] for aiming devices, and video surveillance. In image matching, there are essentially two images of the same scene but with different scales, angles, and perspectives. Given a first pixel in the first image (source image), the goal of image matching is to find the matching pixel in the second image (target image). In some application scenarios, such as navigation and virtual training, video-to-image matching and video-to-video matching are also required. Essentially, these are also image-to-image matching, where either the source image, the target image, or both are a frame(s) extracted from video sequences. The algorithms used for these scenarios are the same as for image-to-image matching, except that there may be some additional dynamic adjustment algorithms involved to maintain relatively constant frame-to-frame processing time and accuracy [3]. This paper reviews state-of-the-art image-matching research on pixel-to-pixel matching between two images, comparing their advantages and disadvantages, and discusses challenges and future research trends. This review is motivated by our intention to share insights and lessons learned from our practical experience with image-matching techniques. We hope that by summarizing recent advances and highlighting common challenges, we can contribute to the community by helping others avoid potential detours and by promoting research and development in this domain.

### 1.2. Applications of Image Matching

Image matching is a core technique in computer vision that enables machines to compare, align, and interpret visual information across different images. This capability has a broad range of applications across multiple fields. In object recognition and classification, image matching allows systems to detect and identify objects in various scenes and under varying lighting conditions and perspectives, making it essential for smart surveillance, quality control in manufacturing, and mobile applications.

In applications involving aiming devices, such as VR, robotic arms, and virtual training, aiming devices rely on image-matching modules to self-identify the aiming point and 6DOF (pitch, roll, and yaw). In augmented reality (AR) and virtual reality (VR), image matching helps align virtual elements precisely with real-world environments by tracking surfaces, markers, or natural features in real time. In robotics and autonomous vehicles, it supports simultaneous localization and mapping (SLAM) [1], enabling robots or cars to navigate unfamiliar environments by matching camera-captured images with stored visual maps (a global understanding of the operating environment).

As deep learning continues to enhance feature extraction and matching accuracy compared to traditional computer vision methods [4,5,6,7,8], image matching is becoming more robust and applicable, even under challenging conditions such as occlusion, deformation, and viewpoint variation. Overall, image matching is a versatile and indispensable tool that aids machines in interpreting and interacting with the visual/physical world in intuitive ways.

### 1.3. Evolution of Image-Matching Algorithms

Historically, image matching has been dominated by handcrafted local feature descriptors such as SIFT [9], SURF [10], and ORB [11], which demonstrated good performance in many practical applications. However, they have limitations, such as sparse feature points and sensitivity to perspective and illumination changes. With the advent of deep learning, the field has seen a paradigm shift toward learning-based methodologies that aim to improve robustness and generalization through data-driven approaches. These methodologies leverage convolutional neural networks (CNNs) and, more recently, transformers and attention mechanisms [12] to achieve more robust feature-point detection and descriptor generation than traditional handcrafted methods. In the past few years, end-to-end pixel-level matching algorithms have been developed to handle stereo image-matching problems.

Despite significant progress, image matching remains challenging, especially in scenarios involving extreme viewpoint changes, repetitive textures, low-texture environments, and stereo matching [13,14,15]. This paper reviews the evolution of image-matching techniques, explores their current state, and discusses open challenges and future research directions.

## 2. Traditional Handcrafted Image Matching

### 2.1. Template Matching-Based Image Matching

The very first image-matching algorithm, template matching [16], focused purely on the intensity values of certain regions in the image. Essentially, the template is a sub-image (known as the template) centered around the given pixel in the source image, and the goal is to find the best match of the template (and, in turn, the matching pixel) within the target image.

**Figure 1:**
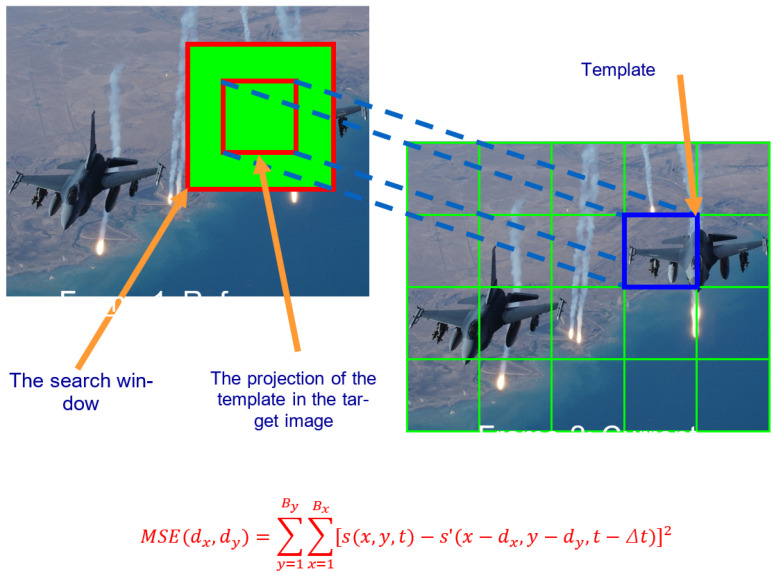
Template-matching process.

Here is how it works: as shown in Figure 1, the template slides over the target image at every possible (x, y) location. Sometimes, in order to reduce computational load, the template only slides within a certain area (also known as the search window) of the target image. At each location, a similarity score between the template and the overlapping region of the target image is computed. Common similarity metrics include cross-correlation, normalized cross-correlation, sum of squared differences (SSD), and Mean Square Error (MSE). The location with the best score is regarded as the matching pixel.

Template matching-based algorithms were the very first effort in the development of image matching and remain straightforward and easy to implement. However, if the search window is large, the algorithms become computationally intensive. A major limitation is their extreme sensitivity to noise, rotation, lighting changes, scale changes, and camera motion. Under these conditions, the algorithms fail. Thus, their practicality is limited, and they can only be used in certain scenarios, such as video surveillance. Nowadays, with the emergence of machine learning-based approaches, template matching-based image matching has become obsolete.

### 2.2. Feature-Based Image-Matching Pipeline

**Figure 2:**
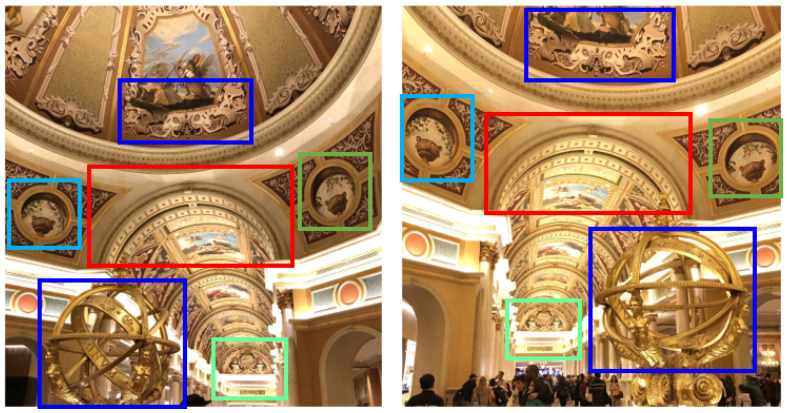
Bounding boxes in the left/right images with the same color represent matching features.

Template matching is the early development of image-matching algorithms. However, it does not naturally simulate the image-matching mechanism used by human vision systems (HVSs). Basically, HVSs do not rely on pixel intensity values. Instead, they rely on features to match a source image and a target image and then establish a matching relationship between them (Figure 2). This has led to the development of feature-based image matching. At its core, feature-based image matching involves detecting and extracting distinctive features from images, such as corners, edges, or textures, and establishing correspondences between them based on the descriptors of the features.

**Figure 3:**
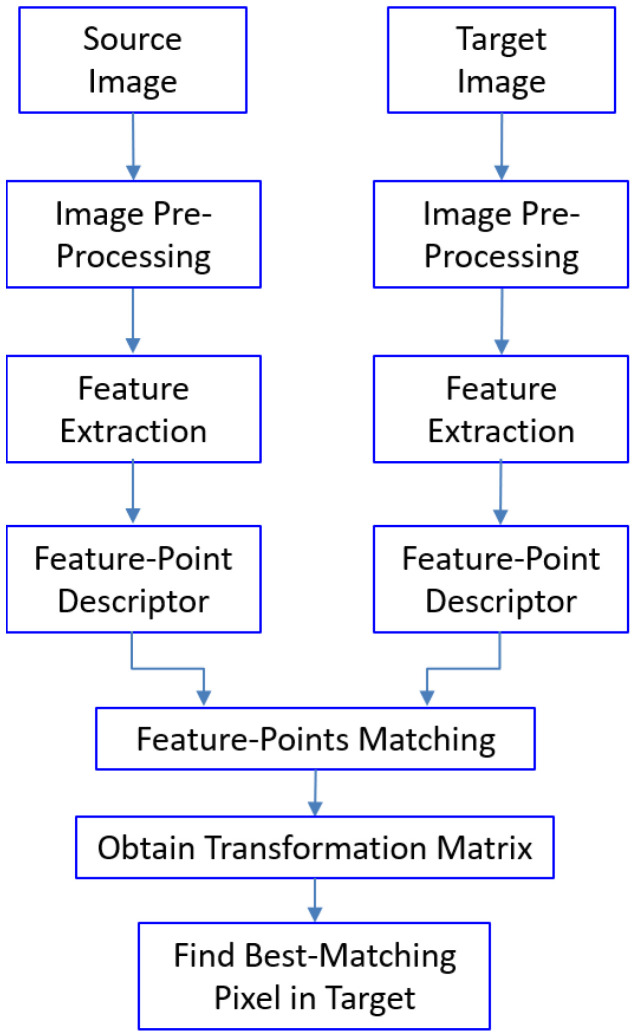
Feature-based image-matching pipeline.

**Figure 4:**
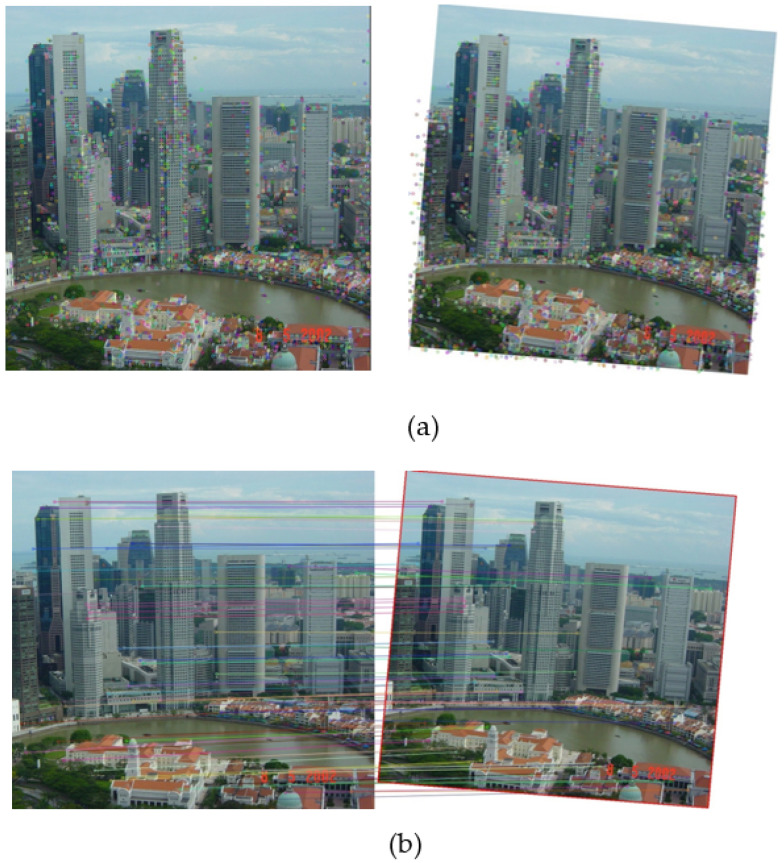
Feature-point extraction (**a**) and matching (**b**).

Figure 3 illustrates a typical pipeline of feature-based image matching. As can be seen, both a source image and a target image are pre-processed (such as downsampling, filtering, or cropping) to prepare for the following steps. The next step is feature-point extraction, which can be handled by a traditional handcrafted feature-matching algorithm (or, more recently, deep learning models). After that, a feature descriptor is extracted for each feature point in both the source image and the target image. The feature descriptor is used to establish the matching relationship between feature points in the source image and feature points in the target image (Figure 4).

The matching relationship, represented by the coordinates of matching feature-point pairs, is used to establish a transformative matching relationship (in the format of a matrix, the so-called homography matrix) between the source image and the target image. The transformation matrix can be obtained and optimized using different algorithms (e.g., the RANSAC [17] or Levenberg–Marquardt algorithms [18]). With the established matching relationship, a given pixel in the source image can be mapped to a pixel in the target image, with a certain level of accuracy. In this framework, each module can be implemented using different algorithms. In the following, the two most successful handcrafted feature-based image-matching algorithms, SIFT (Scale-Invariant Feature Transform) and SURF (Speeded Up Robust Features), are discussed. They both follow the image-matching pipeline outlined in Figure 3 but differ in the algorithms used for feature-point extraction and feature-point descriptor building. The feature-point matching process using the Euclidean distance and the calculation of the transformation matrix (H-matrix) using the RANSAC algorithm are essentially the same. Thus, in the following, only the feature-point extraction and feature-point descriptor building processes are discussed for SIFT and SURF.

### 2.3. SIFT-Based Image Matching

SIFT is the first feature-based algorithm that has achieved wide recognition and adoption. It achieves robustness against image resolution changes and rotation by detecting extrema in an octave-space pyramid using Difference of Gaussians (DoG), then builds a 128-dimensional descriptor for each feature point by analyzing gradient orientations in its local neighborhood.

SIFT builds the octave-space pyramid by downsampling the original image at different resolutions. This allows for reliable detection of feature points at various octave levels, which ensures that the matching algorithm is robust to resolution changes. Within each octave, SIFT progressively blurs the images with Gaussian filters at different scales. After that, SIFT computes the difference between adjacent blurred images to obtain the DoG images. The DoG images highlight areas with sharp intensity changes (edges, corners, blobs), which are ideal candidates for feature points. In the DoG images, SIFT identifies local maxima by comparing each pixel to its 26 neighbors in the 3 × 3 × 3 cube neighborhood—eight neighbors in the same scale, nine in the scale above, and nine in the scale below.

Feature-point detection returns the locations of feature points in both a source image and a target image. The next step is to create a numerical signature (descriptor) for each feature point that describes its local appearance in a way that is robust to changes in resolution, rotation, and illumination. In a 16 × 16 local neighborhood around each feature point, the gradient magnitude and direction are computed. A dominant orientation is assigned to the feature point to make the descriptor rotation invariant. The 16 × 16 neighborhood is divided into 4 × 4 subregions. For each subregion, an 8-bin histogram of gradient directions is generated to capture the intensity change in that area. The 8-bin histogram of all 16 subregions is concatenated and normalized into a 128-dimensional descriptor vector.

### 2.4. SURF-Based Image Matching

Similar to SIFT, the SURF algorithm also follows a two-stage framework: feature-point extraction followed by feature-point descriptor building. However, SURF aims to speed up the process and make it suitable for real-time applications. Like SIFT, SURF finds distinctive feature points that are stable under different scales and rotations but uses less computationally intensive algorithms. Essentially, SURF detects blob-like feature points using the Hessian matrix determinant (computed efficiently with box filters and integral images), then builds a 64-dimensional descriptor by aggregating the Haar wavelet responses in a region around each feature point.

Instead of using Gaussian filtering like SIFT, SURF applies box filters at different scales (approximations of Gaussian filters). Integral images are used to compute the box filters more efficiently. At each pixel and scale, SURF computes the Hessian matrix determinant, which measures blob-like structures. Feature points are selected at locations where the determinant is a local maximum across scales and space.

For each feature point, SURF computes the Haar wavelet responses (approximations of gradients) in the x and y directions within a circular neighborhood to obtain the dominant orientation. Then, a 16 × 16 square region is extracted in alignment with the feature point’s dominant orientation. After that, each region is divided into 16 subregions, each 4 × 4 (as in SIFT). The Haar wavelet responses in all 16 subregions are concatenated and normalized into a 64-dimensional descriptor vector.

Feature-based image-matching algorithms, such as SIFT and SURF, mark a substantial advancement over prior methods. They represent the first attempts to simulate human vision systems in image matching. Instead of relying on pixel intensity values, they focus on features to establish a matching relationship between images. These approaches are robust against rotation, scaling, and perspective changes. However, handcrafted algorithms (SIFT, SURF) are based on fixed rules and mathematical formulas, such as gradients, histograms, and Hessian matrices. They work very well for simple, controlled environments, but they also have the following limitations: (1) they cannot adapt to complex, real-world scenarios (lighting variations, viewpoint shifts, and textureless surfaces); (2) they perform poorly on images without enough features or with repetitive feature patterns; (3) dense and evenly distributed feature-point matching pairs cannot be expected; and (4) the descriptor of a feature point focuses only on local characteristics but lacks position information and global context, leading to false matching in images with repetitive patterns.

## 3. Deep Learning Neural Network-Based Image Matching

SIFT and SURF were the state of the art in the 2000s and early 2010s. But now, deep learning-based methods (like SuperPoint [19], SuperGlue [20], and LoFTR [21]) have largely replaced them in demanding tasks. These new models learn robust features from a large amount of training data and are able to handle much more challenging matching tasks. Also, they are capable of dense matching, which generates a much denser feature-point distribution than SIFT/SURF. The key to the success of these deep learning-based algorithms is twofold: (1) feature extraction is now handled by neural networks that have learned from data (instead of being handled by handcrafted algorithms); and (2) they leverage the self-attention and cross-attention mechanisms of a transformer network to build global feature-point descriptors, replacing the local descriptors built by SIFT/SURF. SuperPoint is a feature extractor, while SuperGlue is a feature-point matcher. They are usually paired together to fulfill the extraction-matching pipeline. On the other hand, LoFTR is known as a detector-less dense matcher. LoFTR does not rely on a feature-point detector but can implement dense image matching by itself.

### 3.1. SuperPoint-Based Image Matching

SuperPoint is a deep learning-based model for feature-point detection and descriptor building, developed by DeTone, Malisiewicz, and Rabinovich in 2018 [19]. SuperPoint was designed to replace SIFT/SURF/ORB and achieve robustness under challenging conditions such as lighting changes, low texture, and image blur, where handcrafted algorithms tend to fail.

The architecture of SuperPoint consists of three main parts: (1) a shared convolutional neural network-based encoder that extracts visual features and builds the feature map; (2) an output head that predicts a heatmap to locate feature points; and (3) another output head that produces a dense map of descriptors. The descriptors are then sampled at the detected feature-point locations to create compact, local representations that are used for image matching.

The training of SuperPoint is implemented through self-supervision instead of manual labeling. It is initially trained on synthetic shapes using a simpler detector known as MagicPoint. Later, it is fine-tuned on real-world images using data augmentation techniques. By applying random geometric transformations (homographies) to real images and enforcing consistency of feature points and descriptors across these transformations, the model learns to generalize well without the need for manually annotated training data.

SuperPoint generates feature-point locations, as well as a local descriptor for each feature point. Compared to handcrafted feature extractors, SuperPoint outputs a much denser feature-point distribution. SuperPoint is a fast and reliable feature-point detector + descriptor model that works well for real-time applications. It is compatible with resource-constrained platforms such as mobile or embedded systems. The model’s reliance on local CNN-based descriptors allows for fast computation, but it also limits its ability to capture global context. It lacks self-attention and cross-attention and thus struggles in highly ambiguous or repetitive scenes where contextual matching is critical. While it outperforms traditional handcrafted methods like SIFT/SURF in both robustness and accuracy, it typically needs to be paired with an attention mechanism-based matcher such as SuperGlue or LightGlue to maximize accuracy in complex environments.

### 3.2. SuperGlue-Based Image Matching

SuperGlue is a deep learning-based method for feature-point matching, introduced by Paul-Edouard Sarlin et al. in 2020 [20]. It was designed to replace traditional feature-matching approaches (such as SIFT/SURF), particularly in challenging visual scenarios such as large viewpoint changes, significant lighting variations, motion blur, low-texture regions, and repeated feature patterns. Its robustness and accuracy in these difficult settings make it a powerful tool for image matching.

Unlike conventional pipelines, SuperGlue is not a feature-point detector or a descriptor builder. Instead, it functions purely as a matcher. It relies on pre-computed feature points and descriptors, most commonly generated by SuperPoint. The strength of SuperGlue lies in its novel matching process, which formulates feature matching as a learnable graph-matching problem. The descriptor of the feature points is enhanced using a transformer-like graph neural network (GNN) that incorporates both self-attention and cross-attention mechanisms. Self-attention allows feature points within the same image to share position and contextual information and build global contextual descriptors. Cross-attention enables interactions between feature points across the two images, modeling their potential correspondences based on both local appearance and relative geometry.

After that, SuperGlue builds a matching score matrix. Each row of the matrix corresponds to a feature point in the source image, while each column of the matrix corresponds to a feature point in the target image. Each cell of the matrix represents the likelihood of a good match between the cell’s row and column. To determine the final matching correspondences, SuperGlue employs the Sinkhorn algorithm (a differentiable approximation) [22], which ensures that the sum of likelihood scores in each row and each column is 1. The algorithm also ensures that each feature point in the source image is matched to at most one feature point in the target image. The output of the Sinkhorn algorithm is a feature-point matching correspondence matrix, along with associated confidence scores for each matching pair.

SuperGlue is a powerful, deep learning-based image-matching framework that enhances feature-point matching through context-aware attention and graph neural networks. Its biggest improvement over prior methodologies is the adoption of a global descriptor. This enables context-aware matching and essentially overcomes the limitations of local descriptors. It delivers superior robustness and accuracy, especially in challenging scenarios (such as repetitive patterns), where handcrafted or local descriptor-based methodologies struggle. However, this advantage comes at the cost of increased computational complexity and memory usage. Additionally, SuperGlue itself is only a matcher. It must be paired with an external feature-point detector and descriptor to implement the full image-matching pipeline.

### 3.3. LoFTR-Based Image Matching

LoFTR (Local Feature Transformer) [21] is a detector-free matching method that builds dense, pixel-level correspondences directly on the coarse-level feature map and refines them through a coarse-to-fine strategy. Being detector-free is the basis of LoFTR’s approach to addressing the repeatability issue of detectors. Traditional detectors have critical weaknesses in areas with low texture and repetitive patterns, such as walls or floors, where localized regions may be very similar in appearance, making it difficult to distinguish them based solely on local information [23]. LoFTR contains four main components:

- The backbone network is constructed using a ResNet [24] variant with an FPN (Feature Pyramid Network) [25] to extract multiscale features from input image pairs. The backbone network can output feature maps of different resolutions at the same time. The coarse-level feature maps, with low resolution and a large number of channels, reduce the length of the sequence to be processed by the subsequent transformer module. The fine-level feature maps, with high resolution and fewer channels, are used for final matching refinement.
- The Local Feature Transformer (LoFTR) module integrates the context of coarse-level features. The coarse-level feature maps obtained from the backbone network are flattened into one-dimensional vector sequences. Positional encoding is added to these vector sequences so that LoFTR can perceive the spatial location information of features. This module consists of four cascaded LoFTR encoder layers. Each LoFTR encoder layer contains a multi-head self-attention layer and a multi-head cross-attention layer. By alternately applying self-attention and cross-attention four times, LoFTR learns globally consistent relationships and outputs transformed coarse-level features.
- The matching module establishes the initial matches. Matrix multiplication is applied to the transformed coarse-level features to compute the score matrix, where each element represents the similarity between features in one input image and another. The confidence matrix is then calculated by applying dual softmax or optimal transport to the score matrix. Reliable coarse-level matches are filtered according to the confidence threshold and the mutual nearest-neighbor rule.
- The fine-level refinement module obtains accurate matches at the sub-pixel level. Coarse-level matches are obtained from low-resolution feature maps with low localization accuracy. For each coarse-level match, its coordinates are first mapped back to the fine-level feature map. Then, projection coordinates are taken as the center, and local windows are cropped to a size of w×w. These fine-level local features are further transformed by a smaller LoFTR module containing one encoder layer. Then, a heatmap of matching probabilities is generated by calculating the correlation between the center feature vector of one window and all the feature vectors of the other window. Finally, the sub-pixel accurate matching coordinates are obtained by calculating the expectation of this probability distribution map.

**Figure 5:**
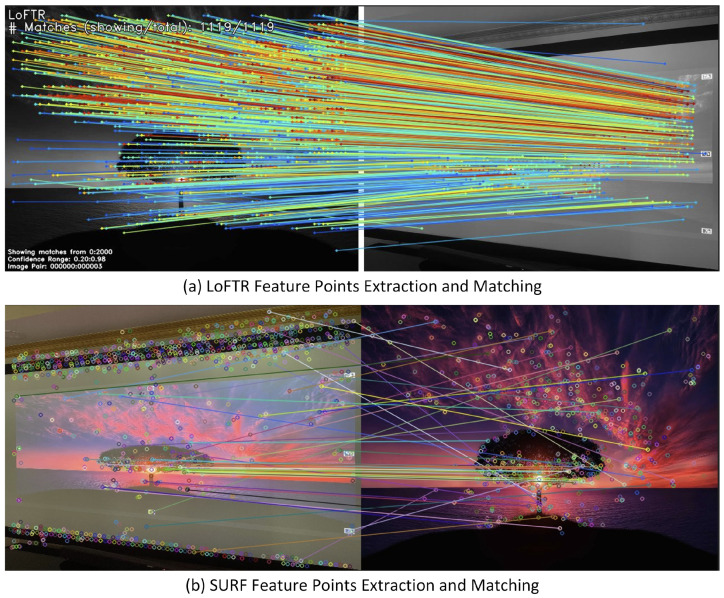
Matching between images with low features: LoFTR (**a**) vs. SURF (**b**).

The advantage of LoFTR is that the entire model can be trained end-to-end without prior feature detection. LoFTR demonstrates strong matching ability in scenes with large viewpoint changes and low texture, and it can produce high-quality matches even in hard-to-distinguish regions such as those with low texture, motion blur, or repetitive patterns. We compared LoFTR with SURF on low-texture image pairs. As shown in Figure 5, LoFTR generated significantly denser and more accurate matching pairs.

Despite the promising performance of LoFTR, its efficiency has been a bottleneck. Due to the need to compute attention on the whole feature map, its computational and memory consumption are very large, limiting its application in real-time, large-scale scenarios. Several methods have been proposed to address LoFTR’s efficiency problem. For example, ELoFTR [26] inherits the powerful matching capability of LoFTR while significantly improving computational efficiency and matching accuracy. The major component redesigns of ELoFTR that contribute to its improvements are as follows:

1. Aggregation attention mechanism: ELoFTR observes that attention computation across all tokens on the feature graph in LoFTR’s transformer module is redundant because neighboring tokens usually contain similar local information. Therefore, ELoFTR introduces an aggregated attention mechanism by adding AG_RoPE_EncoderLayer before each self-attention and cross-attention computation. This is done by applying convolution operations independently to each channel of the coarse-level feature map to generate a learnable query, while the key and query are extracted through max-pooling to achieve spatial downsampling. This significantly reduces the number of tokens involved in the attention computation, thereby dramatically lowering the computational complexity and memory usage. In this way, ELoFTR focuses computational resources on more informative regions while maintaining the advantage of the global receptive field.
2. Efficient backbone network: ELoFTR uses a lightweight RepVGG network as its feature extraction backbone to replace LoFTR’s combination of a ResNet and an FPN. RepVGG uses a multi-branch structure during training to enhance the model’s representational capability, and it can be reparameterized into a simple single-branch structure during inference, thus improving inference speed without loss of accuracy.

### 3.4. LoFTR Speedup

The LoFTR module is the framework’s core component, capturing image dependencies at both coarse and fine resolutions with a transformer architecture. One of the most important components of a transformer-based model is the attention mechanism, expressed by the following equations:

(1) $$\begin{array}{cc} Q,K,V & =\mathrm{Linear}\left(X\right)\\ \mathrm{Attention}\left(Q,K,V\right) & =\mathrm{Softmax}(QK^{T}/\sqrt{d_{k}})V \end{array}$$

where *Q*, *K*, and *V* denote the queries, keys, and values obtained by linearly projecting the input $X\in R^{I\times d}$. The term $QK^{T}$ is the dot product between every query–key pair and forms the attention matrix, whose computation incurs $O(I^{2})$ complexity because both *Q* and *K* contain *I* vectors. Compared with CNNs, dot-product attention offers three key advantages: (a) Global receptive field—Attention explicitly captures dependencies between every pair of tokens, whereas CNNs model only local interactions defined by the kernel size, often requiring deep stacks to achieve a comparable receptive field. (b) Adaptive weighting—The attention matrix assigns data-driven importance weights to tokens, whereas CNNs apply fixed, position-agnostic filters to all pixels. (c) Multi-head attention—By projecting queries, keys, and values into multiple subspaces, multi-head attention allows the model to capture diverse types of dependencies across input tokens simultaneously. It is expressed as follows:

(2) $$\begin{array}{cc} A_{h} & =\mathrm{Attention}\left(Q_{h},K_{h},V_{h}\right)\\ A & =\mathrm{Concat}\left(A_{1},\ldots ,A_{h},\ldots \right) \end{array}$$

where $Q_{h},K_{h},$ and $V_{h}$ are the queries, keys, and values of the *h*-th head.

Although transformers achieve superior performance in feature matching, the quadratic complexity of full self-attention limits LoFTR’s efficiency, especially at the fine level, where input sequences are long. To address this, we introduce two techniques that reduce the complexity to linear time: (a) Sparse attention [27]—Based on the observation that a given image patch is highly correlated with only a small subset of other patches, we restrict each query to attend solely to its most relevant keys. Prior work in image classification and time-series forecasting has shown that sparse attention substantially improves efficiency without sacrificing accuracy. (b) Shifted-window (Swin) attention [28]—An image is partitioned into non-overlapping windows, and attention is applied within each window rather than across the entire image. A subsequent window shift enables information to flow between neighboring windows, preserving global dependencies while keeping sequence lengths short.

We analyzed the computational complexity of LoFTR with the vanilla attention mechanism, Dozer, and the Swin attention mechanism, and the results are presented in Table 1. Dozer attention [27] improved the computational complexity from quadratic to linear, with a coefficient of w+s, where *w* and *s* denote the numbers of keys each query attends to, both far smaller than the input sequence length *I*. Swin attention partitioned the input sequence into multiple windows and applied full attention within each window independently; as a result, the complexity remained quadratic but with a coefficient of 1/m, where *m* is the number of windows (typically set to 4 or 8). By integrating Dozer and Swin attention, we further reduced the computational complexity to linear, with a coefficient of (w+s)/m.

Integrating sparse attention with shifted-window attention markedly accelerates LoFTR, as summarized in Table 2. For this analysis, we fixed the input length at I=4800. When Dozer attention was employed, the LoFTR module’s computational cost fell from 239.32 GFLOPs to 156.76 GFLOPs—a 34.5% reduction—while peak memory usage decreased from 2.981 GB to 2.379 GB. Shifted-window attention further shortened the effective input by dividing the sequence into *m* windows, with m=4 and each window containing 1200 tokens. This configuration reduced FLOPs to 97.71, a 59.17% drop relative to vanilla attention. Combining shifted-window attention with Dozer attention yielded the greatest efficiency: FLOPs were reduced to 76.12 (a 68.19% reduction), and peak memory fell to 0.34 GB (an 88.59% reduction). Because the LoFTR module was applied $N_{f}$ and $N_{c}$ times to the coarse- and fine-level feature maps, these savings accumulated, producing substantial overall efficiency gains. In summary, LoFTR’s efficiency can be dramatically improved by adopting sparsity-aware attention mechanisms.

## 4. Latest Developments in Image Matching

### 4.1. Heavyweight Models

Heavyweight models often require significantly more computing power and are structurally more complex than lightweight models in image matching. For example, the “diffusion” approach has been applied in heavyweight image-matching models, e.g., DiffMatch [29]. By reformulating the matching problem as an iteratively optimized generative task, diffusion models can overcome the inherent limitations of traditional discriminative methods, particularly in texture-less regions and large-displacement scenarios. A common way to apply the “diffusion” concept in image matching is to generate a dense matching field, also known as an optical flow field. The process begins with a completely random, coarse matching field. The model then observes the noisy optical flow field and predicts the noise (i.e., inaccurate matching offsets) present in the current field. By subtracting the predicted noise, the diffusion model produces a more accurate matching field. This iterative denoising process operates globally, allowing information to propagate across the entire image. In texture-less regions, a diffusion model can potentially infer how an entire region should be shifted based on accurate matches established in neighboring areas. Ultimately, diffusion models can generate plausible matches even when features are blurred or occluded, rather than failing outright, as prior methods often do.

Due to their more complex structures, heavyweight models often require larger amounts of training data. To address this issue, researchers sometimes rely on generated data. For example, NeRF-supervised feature matching [30] uses NeRF [31] as a tool to generate high-quality training data that supervises and improves the performance of independent and efficient 2D matching networks. This approach mitigates the problem of training data acquisition in image matching and can provide sub-pixel-level ground truth for pixel correspondences. Even in challenging scenarios where traditional matching methods tend to fail, such as texture-less regions, repeated patterns, or large perspective changes, NeRF can still provide stable and robust training data thanks to its ability to model the underlying three-dimensional geometry. SFD2 [32] tackles the lack-of-data issue with a feature-centered approach that leverages semantic information to generate higher-quality feature points and descriptors. Its core idea is to use semantic segmentation maps to guide feature-point detection, discouraging detection in unstable or uninformative regions (e.g., sky, water) and encouraging detection on semantically meaningful objects (e.g., vehicles, faces). This ensures both the quality and the matchability of the feature points [33]. Unlike other modern methods, such as R2D2 [34], which pursue reliability through a learned repeatability score, SFD2 achieves similar goals through explicit semantic guidance. Furthermore, when generating feature descriptors, SFD2 encodes the semantic information of each point, enhancing its discriminative and analytical capabilities.

Heavyweight models have more complex structures than lightweight models, enabling them to handle more challenging scenarios such as dynamic changes or the need for greater robustness. Conventional lightweight matching methods rely heavily on 2D visual appearance, but they often fail under significant perspective or lighting changes. To address this, RoMa [35] and Matching 2D Images in 3D [36] represent a paradigm shift by elevating the matching problem into 3D space. RoMa focuses on achieving robust dense feature matching, particularly in video scenes with drastic appearance variations (e.g., lighting changes, seasonal differences). Its core technique leverages differentiable rendering, allowing the model to synthesize image appearances under different conditions by predicting both feature maps and depth maps [37]. This enables RoMa to perform self-supervised learning with unprecedented robustness to real-world appearance changes. Matching 2D Images in 3D takes a different approach by finding correspondences directly in 3D space. It predicts the 3D coordinates of original 2D image pixels without relying on depth measurements, while also estimating the feature-point selection probabilities (feature-point distribution) and descriptors that influence the likelihood of matches (matching distribution). By combining these two distributions, the method computes the probability of two feature points being matched within the original 2D images. Similar concepts were explored in the pioneering NeRF-based work BARF [38], which demonstrated that camera parameters can be reverse-optimized through rendering losses.

### 4.2. Lightweight Models

Deep learning models are becoming more and more powerful nowadays at the cost of higher complexity and computational intensity. This requires a large amount of computing power, which may not always be available. For example, in edge devices, mobile applications, and wearable technology, lightweight models capable of real-time execution are highly desirable. This demand has led to a growing focus on lightweight image-matching models within research communities.

LoFTR [21] introduced a novel paradigm by eliminating keypoint detectors and relying on dense feature matching using transformer-based global context aggregation. However, LoFTR’s computational cost and memory usage are relatively high due to the heavy attention operations across dense feature maps. ELoFTR (Efficient LoFTR) [26] is a lightweight and accelerated successor of the original LoFTR model, designed to improve computational efficiency while retaining competitive matching performance.

While LoFTR performs full-resolution attention across dense feature maps, ELoFTR reduces this burden through windowed self-attention and cross-attention within local regions. Additionally, ELoFTR applies early downsampling in the encoder to limit spatial resolution during global context aggregation, and then upsamples selectively to recover spatial detail. The model also replaces some of LoFTR’s heavier modules with lightweight modules, leading to a substantial reduction in floating-point operations (FLOPs), memory usage, and runtime. As a result, ELoFTR achieves comparable accuracy in many benchmarks while being faster and more resource-efficient, making it more suitable for real-time and resource-constrained applications.

LightGlue [39] is a lightweight and efficient image-matching framework that builds upon the principles of SuperGlue. It makes architectural simplifications to reduce complexity, achieve faster performance, and lower resource usage. Similar to SuperGlue, LightGlue performs contextual matching by modeling relationships between local descriptors from source/target images using an attention-based transformer architecture. However, LightGlue adopts a simplified and streamlined design using fewer transformer blocks and optimized attention mechanisms (such as parallel self- and cross-attention). It also incorporates early-stopping criteria during matching inference phases, which helps avoid unnecessary processing when high-confidence matches are already identified. The key advantage of LightGlue is that it preserves much of SuperGlue’s matching accuracy while reducing inference time and memory consumption, making it highly suitable for real-time applications like SLAM, Structure-from-Motion (SfM), and AR on mobile/embedded/edge devices.

In recent years, multiple lightweight vision transformer models, such as MobileViT [40] and TinyViT [41], have been designed for efficiency on mobile and edge devices. MobileViT combines convolutional neural networks (CNNs) with transformers, making it a natural evolution of MobileNet [40] with global reasoning capabilities. TinyViT, on the other hand, was distilled from large-scale transformers, significantly reducing parameters while retaining strong accuracy. Both MobileViT and TinyViT were designed as lightweight models that balance accuracy and efficiency. In short, MobileViT originates from the CNN family and adds transformer capability, while TinyViT was distilled from the transformer family. The model compression strategies of MobileViT and TinyViT can inform the development of lightweight image-matching models. Specifically, TinyViT’s use of knowledge distillation and windowed attention sheds light on how large transformer-based matchers (LoFTR or SuperGlue) could be compressed into compact variants that preserve accuracy while running efficiently on mobile/edge devices.

While most state-of-the-art image-matching models rely on expensive high-end GPU hardware, XFeat [42] addresses lightweight challenges from another angle. Instead of running on a GPU, its goal is to achieve real-time, semi-dense matching on general-purpose CPUs. By tightly controlling the number of network channels, optimizing the convolution module, and designing a novel matching optimization module, XFeat significantly reduces computational effort while maintaining a high-resolution feature map. It demonstrates that superior performance can be achieved without hardware acceleration by using a well-designed and efficient network architecture, providing a plug-and-play solution for resource-constrained devices.

## 5. H-Matrix Optimization

The majority of state-of-the-art image-matching algorithms output feature-point matching pairs between a source image and a target image. However, these algorithms (including the algorithms that claim to be dense matchers) are not dense enough to provide pixel-to-pixel matching. To bridge this gap and meet the requirements of pixel-to-pixel dense matching, we must first build a uniform/global transformation model between the source image and the target image (assuming that all pixels in the source image lie on the same plane, and the same applies to the pixels in the target image). And then, any given pixel in the source image can be mapped to a pixel in the target image using this uniform/global transformation model. This model is called an H-matrix, and it is a 3 × 3 matrix. If point P is on the source image and the H-matrix is known, then P can be mapped to $P^{\prime }$ on the target image using the following equation:

(3) $$P^{\prime }=H\cdot P$$

Various methods can be applied to calculate an H-matrix based on the outputs from a LoFTR model (pairs of feature points between a source image and a target image). This paper compares two methods: (1) the findHomography function provided by the OpenCV library, which uses the RANSAC algorithm [17]; and (2) a genetic algorithm-based method. Since the OpenCV function is widely used, this paper focuses on explaining how the GA-based method works, while skipping detailed explanations of the OpenCV approach.

### 5.1. GA Algorithm-Based H-Matrix Calculation

A genetic algorithm can estimate a homography matrix by evolving the initial randomly generated homography matrices with the goal of minimizing distances between estimated mapping points (i.e., $P^{\prime }$ in Equation (3)) and ground-truth mapping points (i.e., feature points on the target image generated by LoFTR). The steps are detailed as follows:

- Step 1: Randomly initialize N individuals for the first generation. Each individual represents a possible homography matrix.
- Step 2: New individual generation. In each generation, new individuals are generated based on those from the previous generation. This ensures that useful information learned so far is preserved while allowing for new variations. To achieve this, two commonly used functions, crossover and mutation, can be applied. The crossover operation combines information from parent individuals to generate a new individual. The frequency of the mutation operation is usually low (i.e., a small mutation rate), and it updates the information of an individual randomly.
- Step 3: Selection. To ensure that more suitable individuals are generated in subsequent generations, individuals with higher fitness values (i.e., those that reduce the distances between estimated mapping points and ground-truth mapping points more effectively) are selected, while those with lower fitness values are removed from the current generation. Typically, the number of individuals in each generation is kept constant.
- Step 4: Termination. Steps 2 and 3 are repeated multiple times until the fitness value of the best individual converges (i.e., does not change compared to the best individual from the previous generation).

**Figure 6:**
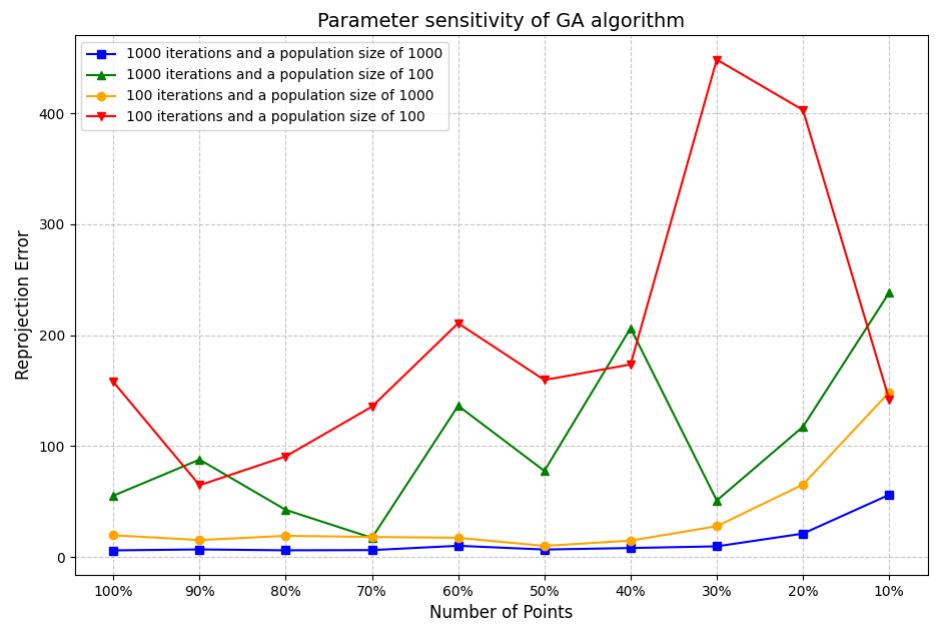
Parameter sensitivity of the GA algorithm.

Figure 6 shows how two key parameters of the genetic algorithm (i.e., the number of iterations and the population size) work together to affect the performance of the algorithm. The population size refers to the number of individuals contained in each generation. A large population can cover a wider search space, increasing population diversity and thus reducing the risk of the algorithm falling into local optimal solutions. For problems that are relatively simple or have limited solution space, we normally work with a smaller population. For complex multidimensional problems or problems requiring high-precision solutions, larger populations are usually required. The number of generations refers to the total number of algorithm evolutions. The algorithms generate new, possibly better populations through operations such as selection, crossover, and mutation in each generation. The number of iterations determines how much time the algorithm has to search and optimize. We set it to 100 for faster verification of the effectiveness of the algorithm. However, the algorithm converged too early, and the quality of the solution improved slowly, so the number of iterations needed to be increased. Increasing the number of iterations to the same population size resulted in a dramatic reduction in the reprojection error; a sufficient number of iterations is essential for the algorithm to converge to an optimal solution, and an insufficient number of iterations leads to very inaccurate inference results. Also, with the same number of iterations, a larger population can lead to a lower error because it provides more genetic diversity, helping the algorithm escape local optima and find a better global solution. Therefore, in order to achieve the desired performance of the genetic algorithm, we set a sufficiently high number of iterations and a sufficiently large population size.

**Figure 7:**
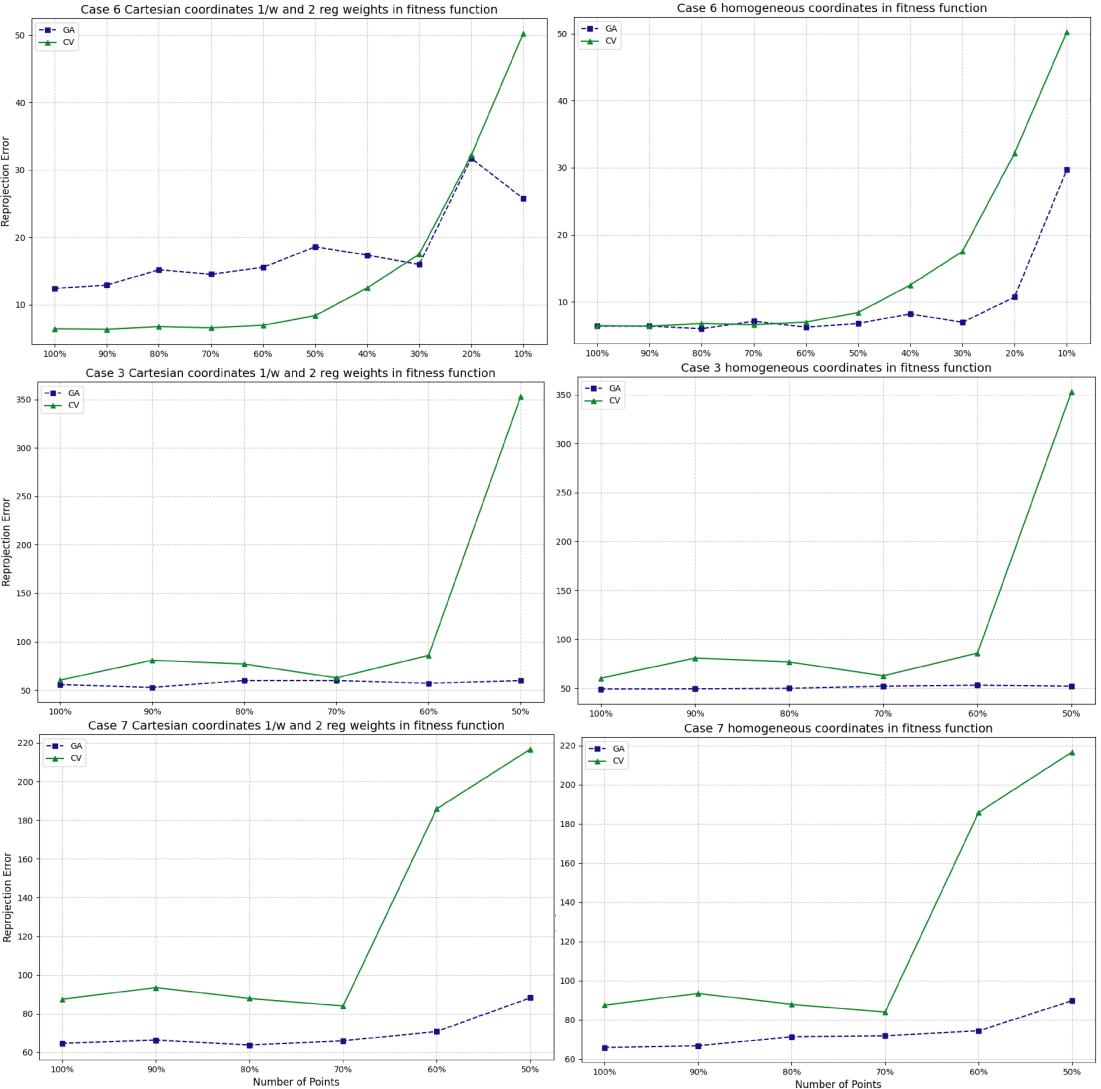
Benchmark performance comparison between findHomography (i.e., CV) and the GA-based method.

In this paper, we conducted experiments to compare the performance of findHomography and a GA-based method (see Figure 7). Case 3 represents stereo image matching, where not all pixels lie on the same plane. Case 6 represents typical 2D images, where both the source and target have all pixels on the same plane. The experimental results showed that the errors from findHomography and the GA-based method varied significantly across different cases (e.g., Case 3 had a much higher margin of error than Case 6). The errors for findHomography (i.e., CV in the figure) tended to increase more significantly and drastically than those of the GA method as the number of points decreased, particularly when the percentage fell below 60% or 50%. This indicates that, under these experimental conditions, the GA method demonstrates greater robustness to reductions in the number of points used for estimation. However, we also observed that the GA-based algorithm can be significantly more time-consuming compared to the findHomography method.

### 5.2. H-Matrix Optimization with the Levenberg–Marquardt Algorithm

The Levenberg–Marquardt (LM) algorithm is widely used for nonlinear least-squares problems. It combines the Gauss–Newton method and gradient descent to minimize an objective function of the form

(4) $$\min_{h}\sum_{i}r_{i}{\left(h\right)}^{2}$$

The update rule is

(5) $$h_{k+1}=h_{k}-{\left(J^{T}J+\lambda I\right)}^{-1}J^{T}r$$

where *h* is the algorithm parameter; *J* is the Jacobian of the residuals; *r* is the residual vector; and λ is a damping factor that smoothly interpolates between Gauss–Newton and gradient descent.

In our context, LM refines the estimated homography between the two images to better align feature points by minimizing the reprojection error. The reprojection error is a key metric that can be used to evaluate the geometric accuracy of feature-point matching. It measures how far a predicted feature point lies from its expected position when projected between images using a transformation (e.g., a homography or essential matrix). In our context, the reprojection error quantifies how well the predicted correspondences align between the original source image and the distorted target image (captured from a screen at an angle).

Mathematically, it is defined as the Euclidean distance between the predicted feature point $\left(x^{pred},y^{pred}\right)$ and the ground-truth feature point $\left(x^{gt},y^{gt}\right)$:

(6) $$Reprojection\;Error_{i}=\sqrt{{\left(x_{i}^{pred}-x_{i}^{gt}\right)}^{2}+{\left(y_{i}^{pred}-y_{i}^{gt}\right)}^{2}}$$

**Figure 8:**
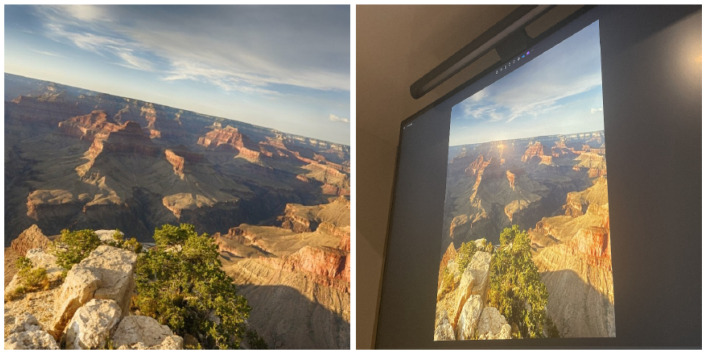
**Left**: Original source image. **Right**: Source image displayed on a monitor and photographed at an angle.

The average reprojection error across all matched points gives a summary of the overall alignment quality. Lower values indicate better geometric consistency between matched images. Experiments were conducted using the ELoFTR model (see Table 3), a simplified, lightweight version of LoFTR with higher execution efficiency [26]. A high-resolution landscape image of the Grand Canyon was used as the source image, and a second image was captured by photographing the source image displayed on a monitor at an angle. This setup introduced realistic projection and perspective distortions, as shown in Figure 8.

We used the two images in Figure 8 as the source and target images. The initial version of the H-matrix was estimated using the RANSAC algorithm. After that, a refined version of the H-matrix was generated by the LM algorithm. The goal of this refinement was to minimize the reprojection error. To evaluate performance, the reprojection error was evaluated before and after LM optimization. As shown in Table 3, the mean horizontal error, mean vertical error, and mean reprojection error were all reduced by significant amounts through LM optimization.

## 6. Current Challenges

### 6.1. Challenges in 2D Image Matching

Image-matching techniques and algorithms have made significant progress over the past three decades. Deep learning-based algorithms have transformed the field, substantially improving the accuracy of image matching. However, there are still many challenges, and current performance does not yet fully meet speed/accuracy requirements in highly demanding application scenarios.

Real-time performance is still often traded for higher levels of accuracy. Traditional algorithms such as SIFT and SURF can meet real-time requirements but lack the desired accuracy. The new AI-based algorithms have achieved a much higher level of accuracy but require significant computing power. They can only run on expensive high-end GPUs if real-time performance is desired. The development of lightweight deep learning models that can meet both accuracy and real-time requirements is imperative.

Image distortion, occlusion, and rotation still pose significant challenges to the accuracy of image matching. Camera distortion degrades image quality, which in turn affects the accuracy of image matching. Even state-of-the-art models (such as LoFTR) remain very sensitive to rotation. The development of an image-matching engine that is more robust to distortion, occlusion, and rotation is highly desired.

In many application scenarios, image matching is implemented on compressed image/video content transmitted over a network. Typically, the compression algorithms used are lossy methods, such as Motion-JPEG, H.264/AVC, or H.265/HEVC. Lossy compression/decompression significantly impairs the accuracy of image matching. How to compensate for the downgraded image/video quality and maintain consistent accuracy between different frames (possibly compressed using different algorithms, IBP frames) is an important research topic worth investigating.

### 6.2. Challenges in Stereo Image Matching

**Figure 9:**
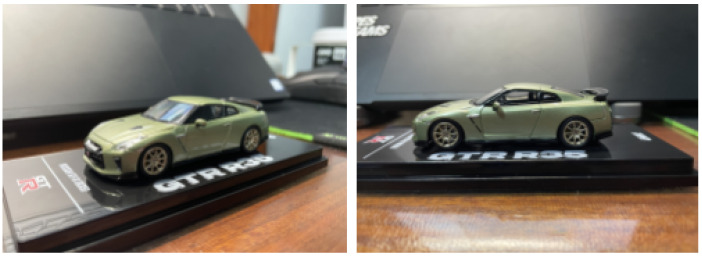
Stereo image matching.

Currently, state-of-the-art algorithms primarily rely on feature point-to-feature-point matching, despite some claims of achieving pixel-to-pixel dense matching. Thus, the matching from any given pixel in a source image to a pixel in a target image still relies on the H-matrix. This paradigm assumes that the transformation relationship between the source image and the target image is linear and uniform and can be modeled using an H-matrix, which may not always hold true. In the two images shown in Figure 9, a 3D scene is captured from two different angles. Some parts of the left image are not visible in the right image, and vice versa. Also, not all pixels lie on the same geometrical plane. In this situation, the transformation from the left image to the right image is not uniform and thus cannot be modeled using an H-matrix. Handling image matching in this scenario (stereo image matching) is a very challenging problem in computer vision. So far, there is no perfect solution for this.

In traditional stereo image matching, most methods assume that the input image pairs are rectified, meaning that matching pixels are on the same horizontal line. This assumption greatly simplifies the matching problem but also significantly limits applicability in real-world scenarios. In practical applications such as drone mapping, virtual reality, and robot vision, stereo image pairs may exhibit arbitrary baselines, varying orientations, and wide viewpoint changes. In such cases, corresponding pixels lying on the horizontal line cannot be assumed, and the standard disparity-based formulation will fail. To address this challenge, some pipelines first estimate the relative pose between images using feature-based matchers like SuperGlue or LoFTR and then rectify the images to prepare them for conventional disparity-based matching. This two-stage pipeline may not always be feasible, and the rectification process could also cause error propagation.

To address unrectified stereo matching, several recent models explore dense pixel-to-pixel matching estimation. Notably, RAFT (Recurrent All-Pairs Field Transforms) [43] estimates a dense optical flow field between two images via iterative refinement and can be repurposed for unrectified stereo matching when viewpoint changes are moderate. Similarly, models like COTR (Correspondence Transformer) [44] predict dense correspondences over the entire image domain using coarse-to-fine or transformer-based attention mechanisms. These approaches bypass the horizontal disparity constraint and are more robust to general viewpoint changes, making them suitable for more challenging scenarios.

Overall, the research trends for stereo image matching include the following: (1) Sparse-to-dense matching, starting from sparse matching (such as LoFTR and SuperGlue) and propagating it to dense matching. This approach combines the real-time performance of sparse matching and the completeness of dense matching. (2) Replacing the traditional pipeline (feature extraction → feature description → matching) with end-to-end neural networks that predict disparity/depth directly from image pairs. (3) Semantic segmentation. This approach segments images into simple regions (such as different planes) to facilitate subsequent stereo image matching. (4) 3D-aware image matching, which constructs a 3D space-aware feature-point descriptor (instead of a 2D feature-point descriptor) to incorporate real-world 3D position information into feature-point descriptors. This approach has the potential to establish a 3D transformation model for stereo image matching (similar to the role of the H-matrix in 2D image matching).

## 7. Conclusions and Future Work

Image matching has long been a significant challenge in computer vision. The methodologies used to achieve this goal have evolved from traditional template matching-based algorithms to feature-based algorithms. In recent years, with the development of deep learning neural network models and attention mechanisms, significant progress has been made in improving the accuracy of image matching, especially in challenging scenarios. In this paper, we have reviewed the typical algorithms and models that have advanced the development of image-matching paradigms. Their features and pros/cons are summarized in Table 4.

In the past few years, the accuracy of 2D image matching has reached a satisfactory level, with the emergence of transformer-based matchers such as SuperGlue and LoFTR. However, stereo image matching remains a significant challenge, and no perfect solution for this scenario has been developed so far. Current research has focused on accurate and robust stereo image matching. Another research trend is the development of lightweight models that can achieve both speed and accuracy in mobile applications, edge computing, wearable devices, and similar contexts.

## Figures and Tables

**Table 1:** Computational complexity of self-attention. The input sequence length is denoted by *I*.

Method	Transformer [12]	Dozer [27]	Swin [28]	Swin + Dozer
Self-Attention	$O(I^{2})$	O((w+s)I)	$O(I^{2}/m)$	O((w+s)I/m)

**Table 2:** Model complexity results for the LoFTR module.

Model	Full	Dozer	Swin	Dozer and Swin
Params (M)	5.251	5.251	5.251	5.251
FLOPs (G)	239.32	152.83	97.71	76.12
Memory (M)	2.98	2.37	0.38	0.34

**Table 3:** Comparison of the impact of LM refinement on feature-point alignment metrics.

Condition	Mean X Error	Mean Y Error	Mean Reprojection Error
With LM Refinement	0.5706	1.1727	1.3838
Without LM Refinement	0.9587	1.1873	1.6236

**Table 4:** Comparison of different models.

	Type	Model Size	Descriptor Type	Accuracy	Computational Complexity	Robustness	Application Scenarios
Template Matching	Handcrafted	N/A	N/A	Low	Medium	Very low	Very limited
SIFT	Handcrafted	N/A	Local	Medium	Fast	Low	Limited
SURF	Handcrafted	N/A	Local	Medium	Fast	Low	Limited
SuperPoint	Learning-based	Large	Local	High	High	High	Broad
SuperGlue	Learning-based	Large	Global	High	High	High	Broad
LoFTR	Learning-based	Large	Global	High	High	High	Broad
ELoFTR	Learning-based	Large	Regional	High	Medium–high	High	Broad
LightGlue	Learning-based	Large	Regional	High	Medium–high	High	Broad

## Data Availability

The original contributions presented in this study are included in the article. Further inquiries can be directed to the corresponding author.
